# Creosote in Pneumonia

**Published:** 1903-03

**Authors:** 


### Creosote in Pneumonia.

Sanitätsrath Dr. C. S. Sewening, of Werther, Westphalia, states, (*Deutsche Aerztezeitung*, Berlin, October 1, 1902,) that the recent numerous communications regarding the employment of creosotal in pneumonia incite him to publish the good results which he has obtained from the drug in some other affections. Amongst others he recounts a case of catarrhal cystitis occurring in a surveyor who had worked for several years in a wet coal mine, and whose urine formed a thick, mucilaginous deposit in the chamber. He was ordered:

℞ Creosotal ........................... 4 grams (1 dram).
Ol. oliv ........................... 200 grams (6⅔ ozs).

After he had taken this mixture two or three times a day in tablespoonful doses for eight days, his urine became permanently clear.

Another case was that of a young farmer, who came to him about a year ago complaining of a dirty discoloration of the face, hands, and the like, being in fact, a good picture of Addison's disease. Sewening prescribed for him:

| | |
|---|---|
| ℞ Creosotal | 4 grams (1 dram). |
| Ol. jecor | 200 grams (6⅔ ozs). |

directing him to take a teaspoonful of the mixture three times daily. After he had taken the medicine twice the spots disappeared, and they have not returned to this day. The man's general condition, also, is perfectly normal.

